# A Molecular Perspective on Sirtuin Activity

**DOI:** 10.3390/ijms21228609

**Published:** 2020-11-15

**Authors:** Carla S. S. Teixeira, Nuno M. F. S. A. Cerqueira, Pedro Gomes, Sérgio F. Sousa

**Affiliations:** 1 UCIBIO/REQUIMTE, BioSIM - Department of Biomedicine, Faculty of Medicine, University of Porto, Alameda Prof. Hernâni Monteiro, 4200-319 Porto, Portugal; cteixeira@med.up.pt (C.S.S.T.); nunoscerqueira@med.up.pt (N.M.F.S.A.C.); 2 Department of Biomedicine, Faculty of Medicine, University of Porto, Alameda Prof. Hernâni Monteiro, 4200-319 Porto, Portugal; pedro.gomes@cnc.uc.pt; 3 Center for Health Technology and Services Research (CINTESIS), University of Porto, R. Dr. Plácido da Costa, 4200-450 Porto, Portugal; 4 Institute of Pharmacology and Experimental Therapeutics, Coimbra Institute for Clinical and Biomedical Research (iCBR), Faculty of Medicine, University of Coimbra, Azinhaga Santa Comba, Celas, 3000-548 Coimbra, Portugal; 5 Center for Innovative Biomedicine and Biotechnology (CIBB), University of Coimbra, Azinhaga Santa Comba, Celas, 3000-548 Coimbra, Portugal

**Keywords:** posttranslational modifications, protein acylation, lysine deacetylases, sirtuins

## Abstract

The protein acetylation of either the α-amino groups of amino-terminal residues or of internal lysine or cysteine residues is one of the major posttranslational protein modifications that occur in the cell with repercussions at the protein as well as at the metabolome level. The lysine acetylation status is determined by the opposing activities of lysine acetyltransferases (KATs) and lysine deacetylases (KDACs), which add and remove acetyl groups from proteins, respectively. A special group of KDACs, named sirtuins, that require NAD^+^ as a substrate have received particular attention in recent years. They play critical roles in metabolism, and their abnormal activity has been implicated in several diseases. Conversely, the modulation of their activity has been associated with protection from age-related cardiovascular and metabolic diseases and with increased longevity. The benefits of either activating or inhibiting these enzymes have turned sirtuins into attractive therapeutic targets, and considerable effort has been directed toward developing specific sirtuin modulators. This review summarizes the protein acylation/deacylation processes with a special focus on the current developments in the sirtuin research field.

## 1. Introduction

Proteins are the structural and functional base of all living organisms. To date, the number of proteins that comprise the human proteome is still elusive. The analysis of the human genome shows the existence of approximately 25,000 protein-coding genes [1]. This would suggest the existence of the same number of proteins. Interestingly, to date, more than 90,000 different human proteins have been identified. This discrepancy has been attributed to three district mechanisms: the alternative splicing of precursor mRNAs, single amino acid polymorphisms (SAPs) and posttranslational modifications (PTMs) [2,3,4]. Together, these modifications raise the complexity of the proteomes by two to three orders of magnitude and help to explain the discrepancy between the complexity of vertebrate organisms and the sizes of their encoded genomes [2,5].

There are two general categories of protein PTM: the covalent cleavage of specific peptide bonds from a protein backbone, which can occur by autocatalytic cleavage or can be catalyzed by a group of enzymes named proteases, and the covalent attachment of a chemical group, which is usually an electrophilic fragment of a co-substrate, to a nucleophilic side-chain residue from the protein. The last reaction can be reversible or irreversible and can be enzymatically or non-enzymatically catalyzed.

It has been estimated that about 5% of the genomes of higher eukaryotes are dedicated to enzymes involved in the posttranslational modification of proteins [2]. Currently, there is a record of more than 300 different PTMs [2,6], and the most common include phosphorylation, ubiquitination, alkylation, glycosylation, oxidation and acylation [5,7,8,9,10,11].

The variability that these PTMs bring to proteins is further amplified by the existence of complex regulatory networks involving both positive and negative crosstalk between the different PTMs. This complex PTM crosstalk is the basis of a protein modification code and has significant importance in the regulation of cellular functions [5,12,13,14].

The most extensively studied PTMs are phosphorylation and acylation [9,11]. Among the array of different possible acylation reactions that may occur, acetylation is the most common (for example, more than 20% of mitochondrial proteins are acetylated [15]) [11,13,16,17,18].

The function of protein acylation reactions depends on the acyl group that is attached. It was proposed that the small acyl groups (formyl and acetyl) may function as recognition elements for protein–protein interactions, while long chains of fatty acids can target proteins to membranes and affect signal transduction [19].

**Figure 1:**
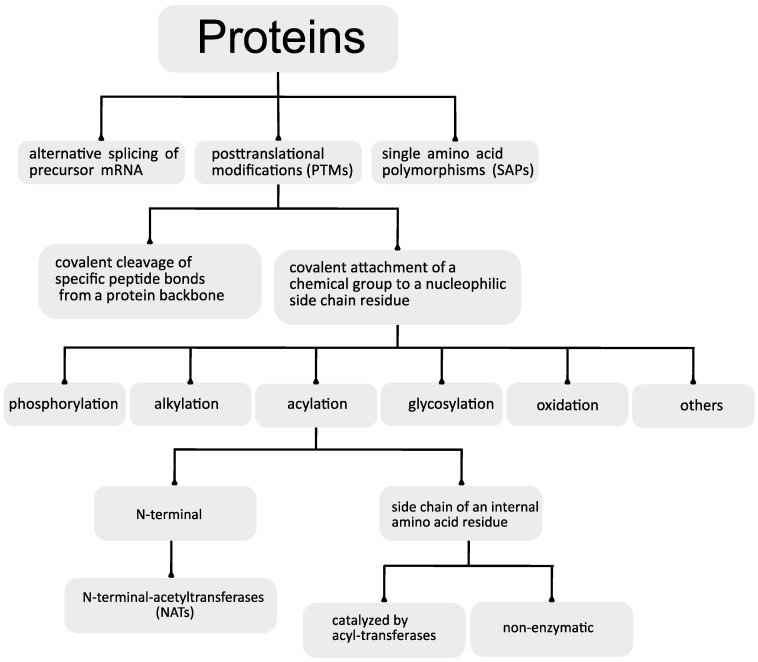
General overview of the cellular events related with the protein acylation process.

**Figure 2:**
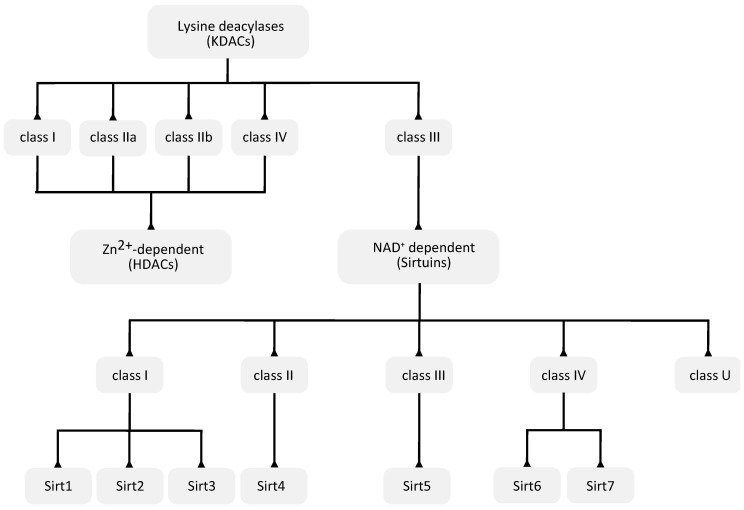
Classification of different lysine deacylases (KDACs) based on their phylogenetic conservation and/or sequence similarities.

The aim of this review is to provide a global overview of the mechanisms underlying both the acylation (Figure 1) and deacylation (Figure 2) of proteins, focusing on a special class of NAD^+^-dependent protein deacylases named sirtuins.

## 2. Protein Acylation

Protein acylation consists of the covalent attachment of acyl groups (acetyl, propionyl, butyryl, malonyl, succinyl, crotonyl, 2-hydroxyisobutyryl, glutaryl, benzoyl, myristate, palmitate, farnesyl, geranylgeranyl, etc.) to amino acid residues bearing nucleophilic side chains, such as lysine (-NH_2_), cysteine (-SH), serine and threonine (-OH), with a specific location in the target protein [19].

The acyl group is generally attached to the side chain of an internal amino acid residue, but acylation can also occur in the protein N-terminus.

### 2.1. N-Terminal Acylation

The known examples of N-terminal acylation are the N-myristoylation of glycine, the N-palmitoylation of cysteine, and N-terminal acetylation [19]. Among those, the most common and best characterized is N-terminal acetylation [20].

#### N-Terminal Acetylation

In eukaryotes, the acetylation of an α-amino group of the N-terminus is an irreversible covalent modification catalyzed by N-terminal-acetyltransferases (NATs) that occurs co-translationally in more than 80% of all proteins [21,22]. N-terminal acetylation neutralizes the positive charge of the free amino group and blocks the N-terminus from further modifications. This modification influences several protein characteristics such as folding, lifetime and degradation, subcellular localization, interactions and complex formation [23,24]. Eukaryotes possess different NATs with different substrate specificities (NatA, NatB, NatC, NatD, NatE and NatF) [25]. Depending on the NAT isoform, the acetyl group may be transferred to the non-cleaved initial methionine residue of the nascent polypeptide chain, or the reaction may occur after the excision of the methionine residue by the ribosomally bound methionine aminopeptidases, and the acetyl group is added to the residue that is positioned immediately after the excised methionine.

### 2.2. Acylation of Internal Lysine Residues

The acylation of internal lysine residues is a reversible and highly regulated PTM that targets many proteins from different cellular compartments, such as the mitochondria, cytosol, nucleus and lumen of the endoplasmic reticulum (ER) [26,27,28]. The target proteins are involved in distinct cellular processes, such as the cell cycle, nuclear transport, chromatin remodeling, mRNA splicing, actin nucleation, signal transduction [26], protein homeostasis and autophagy [27,28].

Although protein acylation can be an activating or inhibitory modification, recent metabolic studies have suggested that, in mitochondria, acylation has a consistently inhibitory role [29,30]

Protein acylation may occur by two distinct mechanisms: it may be catalyzed by acyl-specific transferases that transfer acyl groups from acyl-coenzyme A (acyl-CoA) to the amino acid residues [24,31,32,33], or it may occur non-enzymatically due to the intrinsic reactivity of acyl-CoA thioesters [29,33,34,35].

The first evidence of acylation as a PTM came in 1964 when lysine acetylation was discovered as a PTM of histones [36]. The evidence for the addition of other acyl groups as a PTM was only recently discovered, and therefore, the function and mechanisms behind them are still elusive [7].

#### Acetylation of Internal Lysine Residues

Similar to terminal acetylation, the addition of an acetyl group to an internal lysine residue increases the size of its side chain and neutralizes the positive charge of its amino group. This modification will raise the protein hydrophobicity and consequently induce significant conformational changes that will ultimately affect several protein properties, including transcriptional activity, DNA–protein interactions, subcellular localization, enzymatic activity, folding, peptide–receptor recognition and protein stability [15].

Enzymatic Nε-lysine acetylation has been deeply studied and requires an acetyl group donor (acetyl-CoA), an acetyl group acceptor (the ε-amino group of an internal lysine residue from a polypeptide chain) and an acetyl-CoA:lysine acetyltransferase (lysine acetyltransferase (KAT)) to catalyze the acetyl exchange. The mechanism behind the enzymatic addition of other acyl groups to Nε-lysine requires further studies [29].

Non-enzymatic protein acetylation was identified decades ago [37], and its occurrence was found to be especially favorable in the mitochondria [38,39,40,41,42].

The particular biochemical properties of the mitochondria, namely, its pH and metabolite concentrations, are proposed to favor the occurrence of other non-enzymatic acylation reactions, namely, succinylation, malonylation and glutarylation [29].

### 2.3. S-Palmitoylation of Cysteine Residues

The reversible attachment of palmitic acid to an internal cysteine residue (S-palmitoylation) is the most common type of protein fatty acylation in eukaryotic cells [43,44,45]. The attachment of the palmitic acid to the protein is catalyzed by palmitoyl transferases [46,47], and its removal is mediated by palmitoyl thioesterases.

The palmitoyl transferases belong to the zinc-finger DHHC domain-containing protein family that is characterized by the presence of a conserved aspartate–histidine–histidine–cysteine (DHHC) cysteine-rich domain [46,47]. They are integral membrane proteins, with four to six transmembrane domains and the N and C termini present in the cytosol [48], a characteristic that has made difficult their purification and consequently their identification.

In spite of the recent advances in the field, there are still many questions regarding the functioning of DHHC enzymes. To date, their selectivity, their forms of substate selection and the identification of palmitoylation sites are still elusive [44].

### 2.4. The Influence of Protein Acylation in Human Health and Disease

The attachment of acyl groups to positively charged lysine residues neutralizes the positive charge and consequently affects the protein’s physicochemical properties, ultimately affecting its stability, function, catalytic activity, protein–protein and protein–DNA interactions, degradation and subcellular location [43,44,49].

Many of these post-translationally acylated proteins have relevant roles in vital physiological processes including gene transcription, cell division, cytoskeleton organization, DNA damage repair, DNA replication, signal transduction, protein folding, autophagy, apoptosis, lipid storage and breakdown, mitochondrial fission and fusion, protein synthesis, ion transport, redox and metabolism regulation. [49] Additionally, protein acylation has also been related to the protein aggregation process, which is implicated in several neurological pathologies such as amyotrophic lateral sclerosis [50] and Alzheimer’s disease [51].

Therefore, the correct functioning and regulation of the enzymes involved in protein acylation (and deacylation) are essential for human health, and their misregulation has been associated with several pathologies.

There is evidence of germline mutations in several KATs (for example, KAT6A, KAT6B, CREB- binding protein (CBP) and EP300) that result in disorders, for example, Say–Barber–Biesecker–Young–Simpson (or Ohdo) syndrome [52], Genitopatellar syndrome [53,54] or Rubinstein–Taybi syndrome. These and other disorders caused by KAT mutations are associated with intellectual impairment, developmental delays and physical abnormalities such as facial dysmorphisms [52,53,54,55,56].

Protein acetylation has been associated with several cancers [57], with inflammation and immunity (Falkenberg, 2014, pp. 673–691) and with metabolic diseases such as diabetes [58].

In spite of its evident therapeutic potential, the inhibition of KATs has not been widely explored. A recent study described the development of A-485, a potent and selective catalytic inhibitor of p300 and CREB-binding protein (CBP) that competes with the substrate acetyl-CoA. The compound selectively inhibited cell proliferation in lineage-specific tumor types, including several hematological malignancies and androgen receptor-positive prostate cancer [59].

Another study reported the development of two highly potent and selective inhibitors of KAT6A and KAT6B named WM-8014 and WM-1119, respectively, that are reversible competitors of acetyl-CoA. The inhibition of KAT6A and KAT6B inhibited the growth of lymphoma in mice [60].

Detailed knowledge about the catalytic activity of the different KATs would be important for identifying potent and selective inhibitors for other KATs that would enable obtaining deep knowledge about their biological functions and exploring their therapeutic potential.

Reversible S-palmitoylation is a dynamic process that has several important functions in subcellular protein trafficking, in protein stability (by the prevention of ubiquitination and subsequent degradation) and in the modulation of protein interactions (adhesion and signaling), but its most studied function is its capability to increase the affinity of soluble proteins for lipophilic membranes [61].

The alteration of DHHC expression and the consequent palmitoylation impairment have been associated with several cancers (for example, leukemia, colorectal, hepatocellular and non-small-cell lung cancers) [44,62,63] and with several other disease states resulting from organ-specific processes. One of the most studied cases is the relation between palmitoylation and neuronal functions. There are many reports relating defects in palmitoylation regulation or in the enzymes responsible for palmitoylation and depalmitoylation processes with several neurological disorders such as Alzheimer’s, Parkinson’s or Huntington’s disease, schizophrenia and intellectual disability [44,64].

#### Protein Acylation and “Carbon Stress”

It has been recently suggested that an increase in the concentration of reactive carbon metabolites, resulting from physiological or pathological situations, can culminate in the abnormal occurrence of non-enzymatic protein acylation reactions [29]. This situation can have a detrimental effect on protein function and ultimately disrupt cellular homeostasis. In these scenarios, the non-enzymatic protein acylations are considered a form of “carbon stress” [29].

Recently, it has been found that under carbon stress situations that lead to an increase in protein acylation, a particular group of deacylases, named sirtuins (Sirts), are called to intervene, and, in some conditions, their expression can be upregulated. The sirtuins are part of a protein quality control response that is vital for reducing protein acylation, ensuring protein quality control and consequently reducing carbon stress. An increase in carbon stress and/or the impairment of the carbon stress response will reduce protein quality control and ultimately reduce protein function, leading to several age-related diseases such as neurodegeneration, diabetes, cardiovascular disease or cancer [18,65,66,67,68,69,70].

## 3. Protein Deacylation

Efficient and correct deacetylation activity is of major importance for the correct functioning of several vital biological processes, including DNA recombination [71] and repair [72], transcriptional silencing [73], axonal protection [74], fat mobilization [75], apoptosis [76,77] and aging [78,79].

The hydrolysis of acyl groups from acyl-lysine residues is catalyzed by a group of enzymes named lysine deacetylases (KDACs).

The human genome encodes a total of 18 KDACs that can be grouped into two categories or superfamilies: the Zn^2+^-dependent KDACs or histone deacetylases (HDACs) and the NAD^+^-dependent sirtuin deacetylases (Figure 2).

It should be noted that although grouped in the same family, some KDACs have different acyl selectivities [80,81,82,83], and others are thought to have poor or no deacetylase activity [84].

### 3.1. Zn^2+^-Dependent KDACs

The Zn^2+^-dependent KDACs (or HDACs) are usually called the classic KDACs and account for 11 of the 18 KDACs. They share a highly conserved deacetylase domain and, based on their phylogenetic conservation and in-sequence similarities, can be divided into four classes, named classes I, IIa, IIb and IV (Figure 2). They differ in their enzymatic function, structure, expression patterns and subcellular localization. Classes I and IV are nuclear, class IIb is cytoplasmic, and class IIa is primarily nuclear but upon the activation of signaling is exported to the cytoplasm [84]. Some in vitro experiments suggest that vertebrate class IIa KDACs show poor catalytic activity, which may be related with the replacement of a conserved tyrosine by a histidine in the catalytic pocket [85].

These enzymes exist in multiprotein complexes that determine their substrate specificity, and their catalysis usually generates deacetylated lysine and acetate [49,84,86].

### 3.2. NAD^+^-Dependent Sirtuin Deacetylases

The remaining seven KDACs are named sirtuins and unlike the classic KDACs, they require the cofactor NAD^+^ as a co-substrate [87].

The name sirtuin came from the family’s founding member, the silent information regulator 2 (Sir2) from *Saccharomyces cerevisiae* [88].

They comprise the class III family of KDACs and have seven members (Sirt1–Sirt7). Sirtuins are evolutionarily conserved in all domains of life, and, based on their sequence similarity, they are classified into five classes (I–IV and U) (Figure 2). The seven mammalian sirtuin genes are included in classes I to IV: Sirt1, Sirt2 and Sirt3 are in class I, Sirt4 in class II, Sirt5 in class III, and Sirt6 and Sirt7 in class IV (Figure 2) [89]. Sirt1 has the highest sequence homology to Sir2 in yeast, and it is the most studied sirtuin. The different sirtuins are distinguished by their subcellular localization, acyl lysine substrate specificity, enzymatic activity, and biochemical and metabolic functions. Their impairment is related to different metabolic and health issues [90].

Although deacylation is the main activity catalyzed by the sirtuin enzymes, there is evidence that some sirtuins (Sirt1, 4 and 6) can also ADP-ribosylate protein substrates [91].

#### 3.2.1. Sirtuin Structure

**Figure 3:**
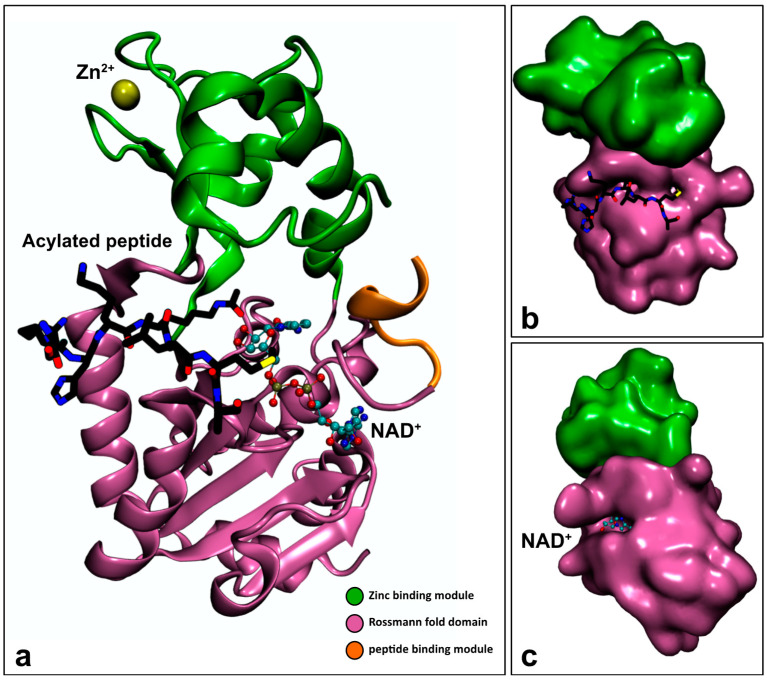
(**a**) VMD representation of the crystallographic structure of a ternary complex from a wild-type Sir2Tm enzyme from *Thermatoga maritima* (PDB ID: 2H4F [97]) (new cartoon representation) with 9 residues from the acetylated p53 peptide (licorice representation), one NAD^+^ molecule (CPK representation) and one Zn^2+^ molecule (VDW representation). The peptide-binding domain (orange) was obtained from the PDB ID structure 2H59 [97] through structural alignment. (**b**) VMD representation of the acylated peptide-binding cleft and (**c**) VMD representation of the C pocket with NAD^+^ inside it.

Sirtuin proteins contain a conserved catalytic core domain composed of approximately 275 amino acid residues flanked by N- and C-terminal regions with a variable sequence and length [78,92]. The catalytic core domain shows a high degree of structural superposition among the different sirtuins. It adopts an elongated shape containing a classical open α/β Rossmann-fold structure that is characteristic of NAD^+^/NADH-binding proteins, and a smaller globular domain composed of two insertions in the Rossmann fold. One of these insertions binds a structural zinc ion that is coordinated with four conserved cysteine residues [93]; the other insertion is a helical module (Figure 3a).

Between the two domains exists a deep cleft where the enzyme active site is located and where both NAD^+^ and acetyl-lysine substrates bind [90]. It was proposed that when the peptide containing the acylated lysine binds to the enzyme’s cleft, its main chain establishes β sheet-like interactions with two flanking strands. One of those strands is positioned in the Rossmann fold. The other one is located in a loop that contains a highly conserved FGExL motif, and is positioned between the Rossmann fold and the Zn^2+^-binding module [94] (Figure 3b). The formation of this so-called “β staple” interaction, in which the substrate links the Rossmann fold and the Zn^2+^-binding module, inserts the acetyl-lysine side chain into a conserved, mainly hydrophobic tunnel and consequently changes the enzyme´s conformation from an open to a closed conformation [93,95]. The closed conformation of the enzyme facilitates the correct binding of NAD^+^ inside a conserved hydrophobic C pocket that is adjacent to the acyl-lysine-binding tunnel. This binding order is important because the occupation of the acetyl-lysine-binding tunnel seems to restrict the binding conformation of NAD^+^ and force it to adopt a “productive conformation” in which its adenine ring forms extensive hydrogen bonds and van der Waals interactions with the Rossmann-fold domain, and its nicotinamide ring is inserted into the C pocket (Figure 3c) [95,96].

#### 3.2.2. Substrate Specificity of Sirtuins

A few studies demonstrate that sirtuins show a high level of substrate specificity for certain acetylation sites in specific substrates [98,99,100]. Accordingly, substrate recognition by sirtuins is affected by differences in the sirtuins’ binding clefts, by the subcellular localization and by some particular characteristics of the substrate, namely, the acylated residue, the attached acyl group, the three-dimensional structure of the substrate, the substrate sequence and the in vivo interactions of the substrate.

##### The Influence of Sirtuin Structure

The sirtuin’s active site is positioned in a cleft composed of the Rossmann-fold domain, the Zn^2+^-binding domain and the four loops that connect the two domains. This cleft is the region of the enzyme that contains the highest sequence conservation within the different sirtuins [98].

The non-Zn^2+^-binding module from the small Zn^2+^-binding domain is the area that shows more variability, either in the primary sequence or in the secondary and tertiary structures among the different Sir2 homologues. This observation suggests that this domain may be involved in the sirtuin’s substrate specificity. Additionally, it has also been observed that the small Zn^2+^-binding domains of the archaeal and bacterial sirtuins have a similar overall topology, while, in eukaryotic sirtuins, they show a higher secondary structure variability [90]. This difference may reflect the higher number of sirtuins that are expressed in eukaryotes and that are required to distinguish a greater number of substrates [101].

Other regions distanced from the active site may also be involved in the sirtuin’s discrimination between different substrates [90].

##### The Influence of the Subcellular Localization of Sirtuins

The different cellular compartments have different proteins; therefore, the distinct subcellular localizations of the seven sirtuins play an important role in their substrate specificities.

Sirt1 is localized predominantly in the nucleus but was also found in the cytoplasm [102]. Sirt2 is mainly cytosolic but was also found in the nucleus [103]. Sirt3 is predominantly localized in the mitochondrial matrix [104], but there is evidence that it moves from the nucleus to mitochondria during cellular stress [105]. Sirt4 was only detected in the mitochondrial matrix. Sirt5 is predominately mitochondrial but is also active in the cytosol [106]. Currently, Sirt6 and Sirt7 are thought to be nuclear.

##### The Influence of the Acylated Residue

The existing data suggest that sirtuins only have deacylation activity on acylated lysine residues [107]. However, since our knowledge of their activity is still very limited, the possibility that they can also deacylate other residues should not be excluded.

##### The Influence of the Acyl Group

In vitro studies showed that only class I sirtuins (Sirt1, 2 and 3) have robust deacetylase activity, although they could also remove long-chain fatty acyl groups. Sirtuins 4–7 have preferences for longer acyl chains [108,109].

Sirt4 was recently shown to remove methylglutaconyl, hydroxymethyl and methylglutaryl from lysine residues [83].

Sirt5 has robust lysine desuccinylase, demalonylase [80,81] and deglutarylase [82] activities. Sirt6 has efficient lysine depalmitoylase and demyristoylase activity [110]. Sirt7 was shown to act as a lysine desuccinylase [111].

Many of these studies were performed in vitro and, therefore, require in vivo validation. The recent development of new methodologies for exploring protein acylation in vivo may bring a new breath of life to this field [19,112].

##### The Influence of Protein Structure

Previous studies suggested that sirtuins deacetylate lysine residues located in regions without a defined secondary structure or in loop regions of the substrate proteins [113]. Recent studies demonstrated that sirtuins are also able to deacetylate lysine residues located in structured or rigid regions of a protein [114]. One of the major limitations in this field is the difficulty of obtaining the co-crystallized structures of sirtuins with natively folded proteins. Indeed, most studies use small peptides containing acetylated lysine residues to study the enzyme–substrate interaction, an approach that may preclude some important information or even skew the conclusions. To get a full picture of the enzyme–substrate interaction, it would be necessary to increase the number of studies using a structural and functional approach in a context of site-specifically acetylated full-length and natively folded substrate proteins.

##### The Influence of Protein Sequence

Structural data show that during catalysis, the side chains of the residues preceding and proceeding the acyl lysine interact with both the Rossmann fold and Zn^2+^-binding module in a “β staple” interaction, as described in the previous section. In order for this enzyme–substrate interaction to be possible and energetically favorable, the side chains of the substrate residues that interact with the enzyme must be chemically and geometrically compatible with the residues that compose the binding cleft of each sirtuin. Because the binding cleft from each sirtuin presents some specific features that distinguish it, the existence of some sequence similarities among the substrates preferentially catalyzed by a given sirtuin would be expected [98,115]. Although some studies have suggested that some sirtuins have a preference for certain amino acid residues in determined sequence positions [98,99,100,114], the results between different studies are not always convergent in their conclusions. A study that performed an analysis of sequences of biochemically confirmed substrates for Sirt2 or Sirt3 concluded that there was no clear consensus in their sequence [93]. On the other hand, some data suggest that the deacetylase activity of Sirt6 is sequence-dependent [110]. In a recent review, it was suggested that this chemical interaction between the enzyme-binding pocket and the substrate residues flanking the acyl lysine could be more important for substrates whose acyl groups show weaker binding affinities for the sirtuin. For acyl groups that bind more tightly to the enzyme´s catalytic pocket, the chemical contribution of those substrate residues would be less important [93].

##### The Influence of Protein Interactions

Under physiological conditions, sirtuins can interact with other proteins or with DNA, and those interactions influence the sirtuin’s substrate specificity. For example, Sirt1 and Sirt3 bind so tightly to their substrate proteins p53 and AceCS2, respectively, that they coimmunoprecipitate [116,117]. In other cases, the recruitment of a sirtuin for a given substrate or vice versa may be mediated by the interaction with other proteins (e.g., transcription factors). For example, Sirt6 and Sirt7 bind to certain transcription factors, which recruit them to different chromatin regions, where they catalyze the deacetylation of a specific histone at specific target genes [118,119,120].

#### 3.2.3. Catalytic Mechanism of Sirtuin Deacylation

According to the existing data, sirtuins are catalytically active only when the peptide containing the acylated lysine is correctly positioned inside the binding tunnel, the enzyme is in the closed conformation, and NAD^+^ has its nicotinamide ring inside the hydrophobic C pocket and the α face of its N-ribose ring exposed to the acetyl lysine carbonyl group [95,96].

**Figure 4:**
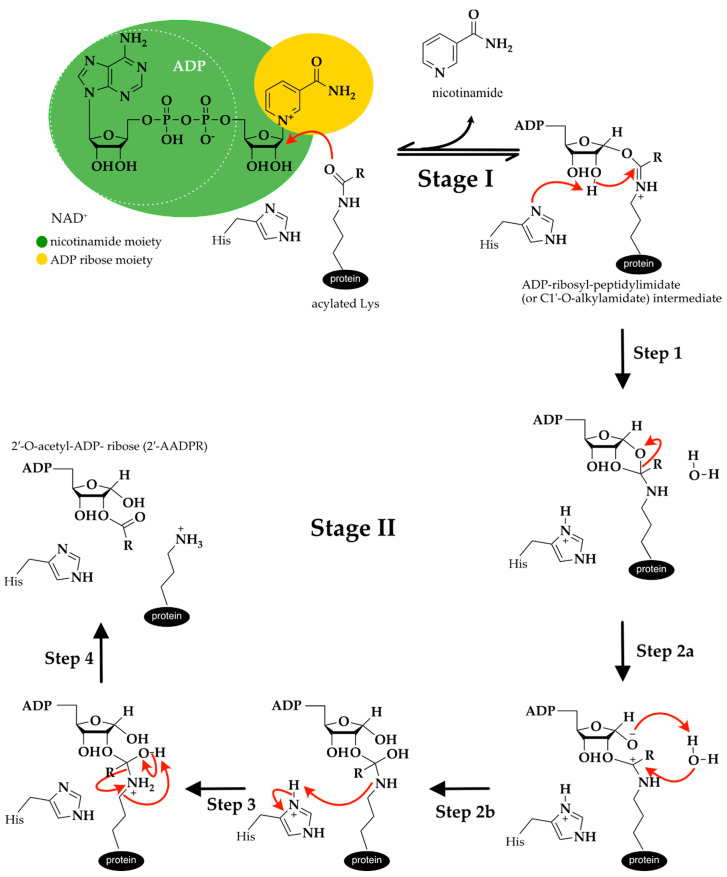
Sirtuins’ catalytic mechanism proposal [122]. The red arrows represent the movement of electrons.

Although it is well established that the general sirtuin catalytic mechanism proceeds in two consecutive stages (Figure 4), the details about the chemistry involved in the generation of each reaction intermediate are still not consensual [121,122].

In Stage I occurs the ADP-ribosylation of acetyl lysine, which involves the cleavage of the nicotinamide moiety of NAD^+^ and the nucleophilic attack of the side chain of the acetylated lysine from the protein substrate to form a positively charged ADP-ribosyl-peptidylimidate (or C1′-*O*-alkylamidate) intermediate and nicotinamide. It was proposed that this reaction occurs through a highly dissociative and concerted displacement mechanism [121]. This reaction is reversible, so NAD^+^ can be resynthesized when nicotinamide concentrations are elevated in solution.

Computational and experimental evidence has shown that Stage II starts with the deprotonation of the 2′-OH group by a conserved histidine residue that acts as a general base (Step 1 from Figure 4). This facilitates the intra–molecular nucleophilic attack of the 2′ hydroxyl onto the positively charged iminium carbon and culminates in the formation of a bicyclic intermediate. In the second step, which is the reaction-limiting step, occurs the collapse of the bicyclic intermediate, in the presence of a water molecule, generating a tetrahedral intermediate (Steps 2a and 2b in Figure 4). In the third step, there is a proton transfer from the positively charged histidine to the amino group of the tetrahedral intermediate (Step 3 in Figure 4). In the fourth step occurs the breakdown of the tetrahedral intermediate into the reaction products: the deacylated lysine and the 2′-O-acetyl-ADP- ribose (2′-AADPR) (Step 4 in Figure 4) [122,123], which can be non-enzymatically isomerized to 3′-O- acetyl-ADP-ribose.

The reaction products are a mixture of 2′- and 3′-O-acetyl ADP-ribose, nicotinamide and the deacetylated peptide [92,124,125].

#### 3.2.4. The Influence of Sirtuins in Human Health and Disease

The sirtuins are a family of enzymes that target different proteins, including histones, transcription factors or proteins involved in DNA repair. Their variable subcellular distribution and the variability of the substrates allows them to control several vital molecular pathways that are involved in cell survival, neuronal signaling, energy metabolism, tissue regeneration, DNA repair, inflammation or circadian rhythms [94,103,118,119,126,127,128,129]

Caloric restriction is the only effective way to naturally extend lifespan and eventually health span in several organisms including humans [130].

Several studies suggest that caloric restriction increases the expression levels of sirtuins, with the exception of Sirt4 [131,132,133] This relation between sirtuin activation and increased lifespan has suggested that sirtuins may have a role in the beneficial effects elicited by a caloric restriction diet [134]. This assumption has boosted the search for potent sirtuin-activating compounds (STACs) [135].

The abnormal activity and/or expression of several sirtuins has been correlated with several cancer types. Several studies suggest that Sirt1 can act both as a tumor promoter and as a tumor suppressor [136,137]. It regulates many tumor suppressors and DNA repair genes, and its upregulation was corelated with a higher chance of being resistant to chemotherapy [138].

Sirt2, similarly to Sirt1, also has a regulatory function, and it has been suggested that it can act both as a tumor promoter and as a tumor suppressor [139].

Sirt3 was shown to act as a tumor suppressor by inhibiting glycolysis metabolism through the deacetylation and consequent activation of pyruvate dehydrogenase [140], but it is also possible that it can act as a tumor promoter in some situations.

Sirt4 was correlated with the inhibition of the progression of colorectal cancer, and its underexpression was correlated with a worse prognosis [141].

The overexpression of Sirt5 in non-small-cell lung cancer tissues was found to be a marker of low survival [142].

Sirt6 showed a controversial role in several cancers. A reduction in Sirt6 expression was correlated with tumor progression with a poor clinical outcome. Its overexpression was shown to promote oncogenic activity in solid and in hematologic tumors [143].

The overexpression of Sirt7 has been associated with aggressive cancers and low survival, whereas its depletion has been associated with a less aggressive phenotype [120].

Sirt1 is the most studied sirtuin. Alterations of the level of Sirt1 expression were associated with the outcomes of several metabolic and neurodegenerative diseases, cancer and aging. A reduction in Sirt1 expression has been related to cardiovascular and neurodegenerative diseases such as Alzheimer’s and Parkinson’s, and with some metabolic diseases such as obesity and diabetes [144,145,146,147,148]. It has been proposed that the downregulation of Sirt1 along with the disease progression may result from the concomitant increase in oxidative stress and inflammation [146,149].

Some age-related diseases and endocrine system dysfunctions are associated with an increase in Sirt1 expression, albeit with a decrease in its activity. It has been hypothesized that in these cases, the increase in Sirt1 expression is a way to compensate for the decline in Sirt1 activity [144].

The current knowledge about the relationships between sirtuins and certain health and disease conditions strongly suggests that the development of molecules capable of selectively activating each human sirtuin variant may bring health benefits through the stimulation of its anti-inflammatory, cardio-protective, neuroprotective and anti-tumor activities. On the other hand, the relationship between the overexpression of some sirtuin variants and the proliferation of certain cancer cells and the development of some metabolic disorders also suggests that their selective inhibition would also be beneficial in certain disease conditions. Therefore, several activators of Sirt1 and inhibitors of Sirt1 and Sirt2 have been developed and are actually in clinical trials. This issue has been deeply explored in a recent review [150].

## 4. Conclusions

Although it is now evident that protein acylation is a complex PTM that embraces the addition of a wide range of different acyl groups to specific protein residues, both the acylation and the deacylation processes require deeper investigation. The major limitation of previous studies is the lack of efficient methodologies that are capable of providing an unbiased identification of the acylated proteins, their acylation modification sites and the specific acyl modifications in vivo. As a significant portion of the current knowledge about these mechanisms arose from in vitro studies, it is important to validate those results in vivo both to prove their biological occurrence and to correlate those modifications with their function in the cell and ultimately in the organism.

The correct functioning and regulation of the enzymes that catalyze the addition and the removal of the acyl groups are of major importance for a variety of metabolic processes, and their impairment has already been related to several human pathologies, including neurodegeneration [64,151,152], cancer [62,63,153,154] and cardiovascular diseases [155,156].

The seminal discovery that the upregulation of the Sir2 gene was able to increase the replicative lifespan of yeast [79,157] has sparked great interest in sirtuin biology. From there on, several studies have shown that sirtuins play critical roles in epigenetics, cell death and lifespan regulation [58,127,128,158] and that their abnormal activity is implicated in several diseases, such as cancer, neurodegenerative disorders, obesity and diabetes [129,159].

Recently, it was found that increasing their activity was associated with the delay of some age-related cardiometabolic diseases [160] and could even increase longevity [161,162,163,164]. These findings have turned sirtuins into attractive therapeutic targets, and considerable effort has been directed toward developing specific sirtuin activators and inhibitors. A deep knowledge of sirtuin enzymatic activity and allosteric regulation is imperative for the development of highly specific mechanism-based sirtuin modulators.

The determination of the catalytic mechanisms of acylation and deacylation by each enzyme, and the identification of the specificities that distinguish them among the members of the same families would be of great importance for improving the current knowledge about these enzymes and the associated enzymatic processes. The knowledge of the transition state structures of the rate-limiting steps of each reaction, with atomistic detail, would provide a promising approach for the design of potent and specific molecules with activating or inhibitory characteristics.
